# Potential adaptive divergence between subspecies and populations of snapdragon plants inferred from *Q*_ST_–*F*_ST_ comparisons

**DOI:** 10.1111/mec.15546

**Published:** 2020-07-24

**Authors:** Sara Marin, Anaïs Gibert, Juliette Archambeau, Vincent Bonhomme, Mylène Lascoste, Benoit Pujol

**Affiliations:** ^1^ PSL Université Paris EPHE‐UPVD‐CNRS USR 3278 CRIOBE Université de Perpignan Perpignan Cedex France; ^2^ Laboratoire Évolution & Diversité Biologique (EDB UMR 5174) Université Fédérale de Toulouse Midi‐Pyrénées CNRS Toulouse France; ^3^ BIOGECO INRA University of Bordeaux Pessac France; ^4^ Institut des Sciences de l'Évolution (ISEM) Montpellier Cedex France

**Keywords:** altitudinal gradient, *Antirrhinum majus*, local adaptation, quantitative genetics, subspecies divergence

## Abstract

Phenotypic divergence among natural populations can be explained by natural selection or by neutral processes such as drift. Many examples in the literature compare putatively neutral (*F*_ST_) and quantitative genetic (*Q*_ST_) differentiation in multiple populations to assess their evolutionary signature and identify candidate traits involved with local adaptation. Investigating these signatures in closely related or recently diversified species has the potential to shed light on the divergence processes acting at the interspecific level. Here, we conducted this comparison in two subspecies of snapdragon plants (eight populations of *Antirrhinum majus pseudomajus* and five populations of *A. m. striatum*) in a common garden experiment. We also tested whether altitude was involved with population phenotypic divergence. Our results identified candidate phenological and morphological traits involved with local adaptation. Most of these traits were identified in one subspecies but not the other. Phenotypic divergence increased with altitude for a few biomass‐related traits, but only in *A. m. striatum*. These traits therefore potentially reflect *A. m. striatum* adaptation to altitude. Our findings imply that adaptive processes potentially differ at the scale of *A. majus* subspecies.

## 1 INTRODUCTION

Local adaptation ‐ the evolutionary response to selection that makes populations fitter in their own local habitat than in other populations' local habitats ‐ is widespread in both plant and animal species (Halbritter et al., 2018; Kawecki & Ebert, 2004; Leinonen, McCairns, O'Hara, & Merilä, 2013). There is evidence for its role in the adaptive divergence of plant species (Halbritter et al., 2018; Hereford, 2009; Leimu & Fischer, 2008). For example, empirical studies have demonstrated differential adaptation in plant sister or hybridizing species, for instance between pairs of *Silene* (Favre, Widmer, & Karrenberg, 2017), *Senecio* (Abbott & Brennan, 2014), and *Mimulus* (Angert & Schemske, 2005) species. These studies compared local adaptation for sister species confronted with different ecological requirements; moister and more fertile versus drier and disturbed sites for *Silene* species (Favre et al., 2017), at high versus low altitude for *Senecio* species (Abbott & Brennan, 2014) and *Mimulus* species (Angert & Schemske, 2005). Different species may also respond similarly to the same type of environmental gradient. Recently, Halbritter et al. (2018) combined the information from studies of multiple plant species along elevation gradients. They found significant evidence for adaptation to different elevations in terms of survival and biomass, with a lower survival at foreign elevations, and a clear trend towards smaller plants at higher elevation. Their results also showed variation across‐ and within‐species in plant responses to elevation. For example, in *Capsella bursa‐pastoris*, native plants from higher elevation flowered at different times, both earlier and later, than plants from lower elevation (Neuffer & Hurka, 1986). The study of local adaptation in populations of closely related taxa exposed to environmental gradients, e.g., altitude, is an opportunity to investigate the conditions promoting or impeding the consistency of adaptive responses.

An indirect approach to investigate whether local adaptation might potentially be involved in the phenotypic divergence of populations is the *Q*_ST_–*F*_ST_ comparison (McKay & Latta, 2002; Merilä & Crnokrak, 2001; Spitze, 1993). The comparison of population genetic differentiation estimated for putatively neutral molecular markers with the population quantitative genetic differentiation estimated for phenotypic traits can be used to identify candidate traits playing a role in local adaptation (Whitlock, 2008). This is done by estimating whether trait quantitative genetic differentiation among populations is more likely the result of divergent selection (*Q*_ST_ > *F*_ST_), stabilizing selection (*Q*_ST_ < *F*_ST_), or neutral evolutionary divergence (*Q*_ST_ = *F*_ST_, e.g., as a result of drift). Some debate around the accuracy of *Q*_ST_–*F*_ST_ comparisons resulted in a variety of methodological adjustments (Edelaar, Burraco, & Gomez‐Mestre, 2011; Ovaskainen, Karhunen, Zheng, Arias, & Merilä, 2011; Whitlock, 2008; Whitlock & Gilbert, 2012). In plants, reciprocal transplants directly comparing fitness between native and non‐native habitats are often preferred to *Q*_ST_–*F*_ST_ approaches conducted in common gardens because they evaluate the effect of environmental conditions (Angert & Schemske, 2005; Etterson, 2004; Kim & Donohue, 2013). When reciprocal transplant experiments cannot be easily undertaken, *Q*_ST_–*F*_ST_ comparisons represent an opportunity for exploring local adaptation hypotheses.

Here, we investigate patterns of local adaptation in two closely related plant subspecies of Snapdragon (*Antirrhinum majus* L., Plantaginaceae) by using *Q*_ST_–*F*_ST_ comparisons estimated in a common garden experiment, and evaluate whether altitudinal gradients might play a role in the potential adaptive divergence of populations. We studied eight populations of magenta‐flowered *A. m. pseudomajus* and five populations of yellow‐flowered *A. m. striatum* sampled along altitudinal gradients. These two subspecies are interfertile (Andalo et al., 2010). They are distributed parapatrically, with the geographic range of *A. m. striatum* surrounded by the range of *A. m. pseudomajus*, and come frequently into contact at the margins of their ranges where there is evidence for gene exchange (Khimoun et al., 2011; Ringbauer, Kolesnikov, Field, & Barton, 2018). Their geographic separation is not explained by actual climatic differences, as illustrated by the substantial overlap of environmental conditions between the two subspecies (Khimoun et al., 2013). This system is therefore promising to explore potential differential adaptive responses of closely related subspecies, in particular regarding the role played by altitude in adaptive divergence.

There is poor support in the literature for adaptive changes in reproductive traits along altitudinal gradients (Halbritter et al., 2018). In contrast, adaptive differentiation is expected for biomass‐related traits and height, with a trend toward smaller plants at high altitude compared to plants at lowland sites (Halbritter et al., 2018). We tested this hypothesis for four morphological traits (basal stem diameter, number of branches on the plant, number of vegetative nodes on the main stem, and total height of the plant). We also studied three additional traits: a phenological trait (germination date), a developmental trait (average internode length) and a functional trait (specific leaf area, SLA). We expected populations from higher altitudes to germinate later and over a shorter period (Donohue, Rubio de Casas, Burghardt, Kovach, & Willis, 2010; Gimenez‐Benavides, Escudero, & Iriondo, 2006). This is because such germination allows plants to track the late arrival and the shorter‐term availability of suitable climatic conditions for growth at higher altitudes (Körner, 1999). Because internode length is a trait related to both plant height and growth rates, we had no clear expectation for how this trait might respond to altitude. Finally, SLA relates to leaf construction cost and captures information about leaf economic strategies (Wright et al., 2004); low SLA suggests high leaf construction cost and high stress tolerance. Selective pressures associated with lower temperatures at higher elevations are expected to promote leaf trait syndromes associated with superior stress tolerance but inferior competitiveness (Read, Moorhead, Swenson, Bailey, & Sanders, 2014). These relationships are generally stronger among species than among populations of the same species (Read et al., 2014). Therefore, we expected either no correlation or a negative correlation between SLA and elevation among populations.

In our study, we estimated neutral genetic differentiation (*F*_ST_), and quantitative genetic differentiation (*Q*_ST_) based on the partition of trait genetic variance and trait heritability (*h* ^2^) in *A. majus*. Previous studies of genetic differentiation between populations and subspecies of *A. majus* at putatively neutral microsatellite markers showed that gene flow was limited between populations (Debout, Lhuillier, Malé, Pujol, & Thébaud, 2012; Pujol et al., 2017), thus favouring adaptive divergence. We tested whether traits were potentially involved with local adaptation by comparing *Q*_ST_ and *F*_ST_, and we investigated whether quantitative genetic differentiation increased with altitudinal difference, and the possibility that environmental changes associated with altitude, which include a suite of climatic variables, drove adaptive responses.

## 2 MATERIALS AND METHODS

### 2.1 Study system

*Antirrhinum majus* L. (Plantaginaceae) is a hermaphroditic, self‐incompatible, short‐lived perennial species, characterized by a patchy distribution in southern Europe centred over the Pyrenees Mountains (Khimoun et al., 2011). It occurs from sea level to an altitude of 1,900 m (Andalo et al., 2010), on limestone or siliceous substrates and in habitats with contrasting moisture regimes (rainfall 500–1,000 mm per year), where it forms restricted stands mostly on rocky outcrops and screes. The species thrives in disturbed habitats, and is especially common along roadsides and railway embankments (Khimoun et al., 2011).

### 2.2 Subspecies of *A. majus*

The two interfertile subspecies of *A. majus*, *A. m. pseudomajus* and *A. m. striatum* produce magenta and yellow zygomorphic flowers, respectively (Andalo et al., 2010). For putative neutral microsatellite loci, they show ca. 1% genetic differentiation (estimated via *F*_ST_), which is one order of magnitude lower than the ca. 10% differentiation found among populations within subspecies (Pujol et al., 2017). There is evidence for gene exchange between subspecies in multiple populations across contact zones (Khimoun et al., 2011). Genome scans across a particular contact zone in the Pyrenees revealed little to negligible differentiation between the two subspecies, with the exception of loci underlying flower colour differences, which were characterized by high differentiation (Tavares et al., 2018; Whibley, 2006). Frequency dependent selection exerted by pollinators on the basis of flower colour is acknowledged to maintain the two subspecies as separate entities (Tastard, Ferdy, Burrus, Thébaud, & Andalo, 2012). The different geographic distributions of *A. m. pseudomajus* and *A. m. striatum* are not explained by habitat differences, as illustrated by the substantial overlap of environmental conditions between the two species (Khimoun et al., 2013).

### 2.3 Collection sites and plant material

**FIGURE 1:**
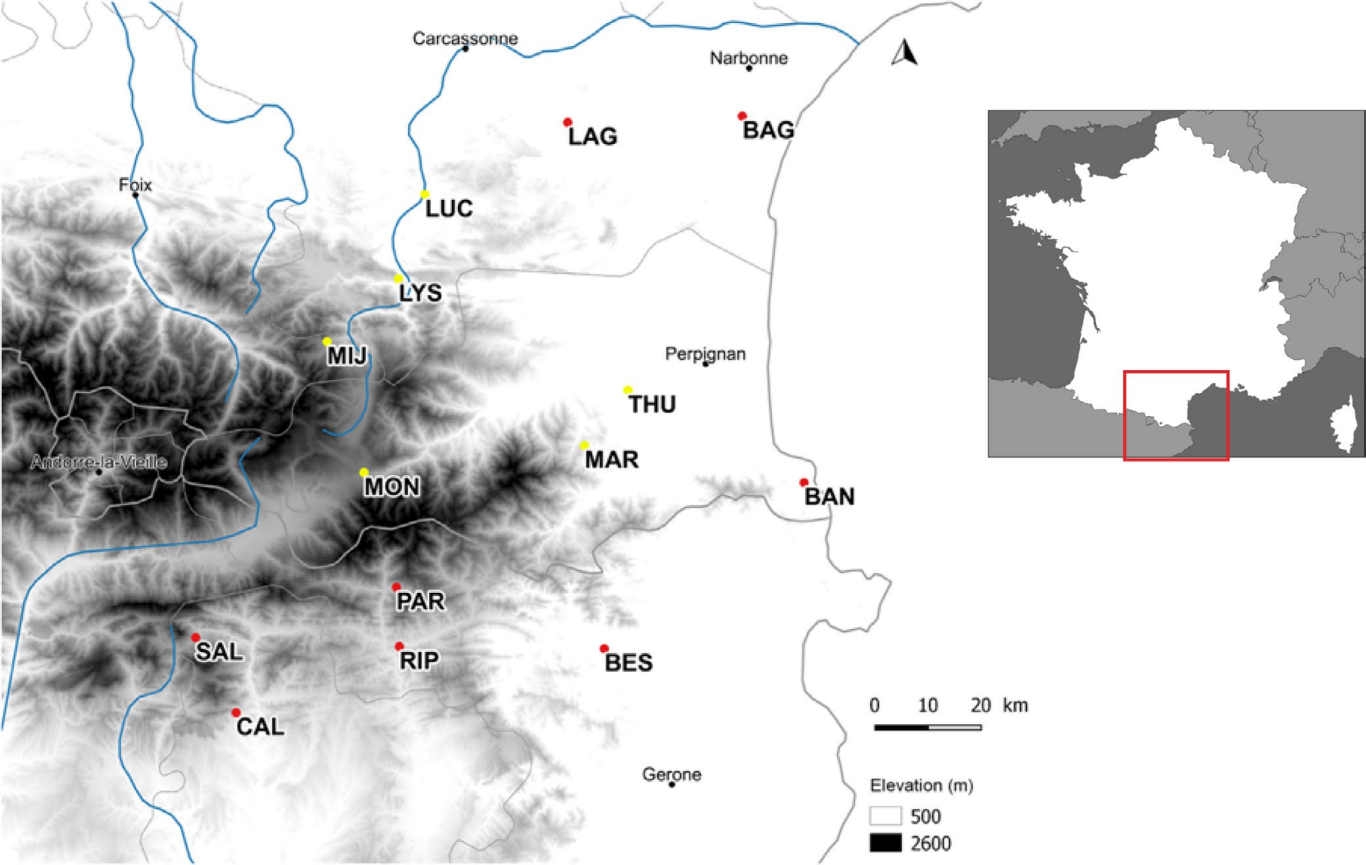
Map of *Antirrhinum majus* populations. *A. majus* populations were sampled across the geographic range of the species in Southern France. Red dots represent *A. m. pseudomajus* populations, yellow dots represent *A. m striatum* populations. Population names and description can be found in Table S1 [Colour figure can be viewed at wileyonlinelibrary.com]

Thirteen wild populations of *A. majus* were sampled in 2011 across its geographic range (between north‐eastern Spain and south‐western France) to represent the overall diversity of the species, with eight populations of *A. m. pseudomajus* and five populations of *A. m. striatum* included (Figure 1; Table S1). For each subspecies, we sampled populations from low and high altitude habitats in different parts of the species geographic range. The variance in altitude was not significantly different between subspecies (see Supporting Information) and should not drive potential differences between taxa. Populations sampled along elevation gradients are likely to be confronted with contrasting environmental conditions. Fifty‐year averages (1950–2000) of mean annual temperature and annual average rainfall were extracted from the WorldClim database (resolution 1 km^2^, www.worldclim.org, Hijmans, Cameron, Parra, Jones, & Jarvis, 2005). They ranged from 14.8°C and 52 mm (at BAN, 61 m above sea level) to 6.1°C and 94 mm (at MON, 1,564 m above sea level; Figure S1). The sampling of populations in different valleys or on different summits limits spatial autocorrelation in the data and shared phylogeographic history between populations from similar altitudes. Thus, populations with similar elevation are not geographically closer.

From each wild population, seeds were randomly collected in October 2011 and used to grow plants in 2012, in a greenhouse at the CNRS Experimental Ecology Station in Moulis, France. Seeds were sown in spring in individual pots (9 × 9 × 10 cm) filled with universal compost. Plants germinated and grew with no nutrient addition under an average temperature from 15°C to 28°C and weekly watering. Mature plants were hand‐pollinated during the summer 2012 to produce progeny from crosses within populations where mates from different families were assigned randomly. Seed of full sib families produced from these plants was stored at room temperature, in the dark, under dry conditions until they were used to produce the plants measured in our experiment. This intermediate generation of plants grown under controlled conditions allowed us to reduce potential maternal environmental effects that could have otherwise affected plants grown from seeds sampled in the wild.

### 2.4 Common garden experiment

Nine to 42 seed families from each of the 13 study populations were grown outdoors in spring 2014 in a common garden at ENSFEA (Toulouse, France). Two plants per family were grown. Some plants died before measurements were made, which resulted in some families being represented by only one plant (Table S1). Plants were grown in individual pots (9 × 9 × 10 cm) filled with universal compost, with no nutrient addition, under outdoor climatic conditions (average month temperatures ranging from 20.6°C to 21.5°C and cumulative monthly rainfall ranging from 28.3 to 73.4 mm). Plants were arranged in a randomized block design (40 plastic containers, 600 × 400 × 120 mm) with each container including 24 randomly chosen plants. Containers of this size were chosen because they optimized the spatial arrangement of pots and the rotation of container locations. Such randomized block design is not expected to inflate artificially differences between populations and families because plants from different populations and families were randomly distributed across several containers. A potential block effect on groups of plants within containers was controlled for at the statistical level. The bottom of each container was covered with an irrigation sheet (400 g.m^−2^) that allowed regulation of the moisture content of the compost. Plants were supplied with water in case of prolonged drought. Damage caused by herbivorous insects was contained by using a wintering veil. This veil also limited pollination.

### 2.5 Phenotypic data

We investigated several vegetative traits on each individual: a phenological trait, a functional trait, a developmental trait, and four morphological traits. The functional trait (SLA) was calculated as the ratio between the cumulated area of five mature but nonsenescent fresh leaves and their oven‐dried mass (Pérez‐Harguindeguy et al., 2016; Pujol, Salager, Beltran, Bousquet, & McKey, 2008). Leaf area was measured by using the R package Momocs v. 1.2.9 (Bonhomme, Picq, Gaucherel, & Claude, 2014). Flower‐related traits were measured but they were not included in this study. This is because not enough statistical power was available to analyse them within subspecies, as less data was available for these traits (not all plants that grew flowered).

### 2.6 Molecular analyses

To infer genetic diversity estimates in each population and to compute *F*_ST_, we genotyped the 637 plants that germinated. DNA was extracted from silica gel dried leaf samples using the Biosprint 15 DNA Plant kit (Qiagen) following the manufacturer's instructions. Individuals were genotyped for 23 putatively neutral microsatellite markers that were developed for population genetic studies (Debout et al., 2012; Pujol et al., 2017). To compute *F*_ST_, we used population pairwise *F*_ST_ estimates and the overall *F*_ST_ estimate amongst populations from the study by Pujol et al. (2017). We used the GenoDive 3.0 software (Meirmans & Van Tienderen, 2004) to compute the complementary parameters required for this study, e.g., the genetic diversity at each locus.

### 2.7 Statistical analysis

All statistical analyses were performed using the R.3.5.0 software (R Core Team, 2018).

#### 2.7.1 Phenotypic traits

First, to test for phenotypic differences between subspecies, hierarchical generalized linear models were conducted with population nested in subspecies. Second, for each subspecies, linear mixed models were conducted to test for phenotypic differences among populations, with population as a fixed effect and the plastic container (“block”) as a random effect. Estimates of marginal means for each trait in each population were extracted using the emmeans package (Lenth, Singmann, Love, Buerkner, & Herve, 2019). These linear mixed‐effects models were implemented in R via the lme4 package (Bates, Mächler, Bolker, & Walker, 2015). Trait changes with altitude were analysed using a linear regression of the marginal means by altitude. Finally, the means for each phenotypic trait were also generated, and provided in the Supporting Information (Figure S1).

#### 2.7.2 Calculation of *h* ^2^ and phenotypic differentiation indices (*Q*_ST_)

For each subspecies, narrow‐sense heritabilities (*h* ^2^) were estimated for each phenotypic trait across all populations using a model with population, family and plastic containers as random factors as *h* ^2^ **=* *2*V*_w_/(*V*_w_ + *V*_res_), where *V*_w_ is the family variance component and *V*_res_ is the residual variance component corresponding to the within‐population variance component. We multiplied *V*_w_ by two in the calculation of *h* ^2^ because we used a full‐sib crossing design (Roff, 1997). Caution must be taken when using this type of *h* ^2^ estimates. Estimates based on full‐sib designs can be less precise than estimates calculated on the basis of a full pedigree. We maximised the precision of our *h* ^2^ estimates by calculating *h* ^2^ based on all the families, without considering the differences of *h* ^2^ between different populations. We also calculated confidence intervals of *h* ^2^ by using a parametric bootstrap method adapted from O'Hara and Merilä (2005).

For each trait and each subspecies, quantitative trait divergence indices (*Q*_ST_) were generated among populations (overall *Q*_ST_) and for each population pair (population pairwise *Q*_ST_) based on mixed model analyses. In these models, population, family and plastic containers were random factors. Variance components were extracted from these analyses for each trait and used for estimating *Q*_ST_ using the following formula (Spitze, 1993): *Q*_ST_ = *V*_b_/(*V*_b_ + 2*h* ^2^ (*V*_w_ + *V*_res_)) with *V_b_* being the trait genetic variance among populations. *h* ^2^ was calculated based on all the families and populations by subspecies. Here, no environmental sources of phenotypic variance due to the ecological conditions of the location of origin of populations could in theory bias *Q*_ST_ estimates because data were obtained from a common garden experiment (Pujol, Wilson, Ross, & Pannell, 2008). When a variance component was nonsignificant, it was considered as null in further calculations. When necessary (as for population pairwise *Q*_ST_ calculation), data were normalized by using a square root transformation. All variance components were estimated by using the linear mixed model approach implemented in the R package lme4 v. 1.1.17 (Bates et al., 2015). Confidence intervals of *Q*_ST_ values were calculated following a parametric bootstrap method adapted from O'Hara and Merilä (2005).

#### 2.7.3 Overall *Q*_ST_–*F*_ST_ comparisons

We compared overall *Q*_ST_ and *F*_ST_ for each trait to investigate if divergence was compatible with a scenario of genetic drift (overall *Q*_ST_ = *F*_ST_), or whether it was more probably explained by directional selection (overall *Q*_ST_ > *F*_ST_) or by stabilizing selection (overall *Q*_ST_ < *F*_ST_). Comparisons between overall *Q*_ST_ and *F*_ST_ values were performed for each trait based on two methods: (a) a comparison of confidence intervals (CIs), the *Q*_ST_ is considered nonsignificantly different from neutral differentiation when the CI of the overall *Q*_ST_ for a trait overlaps the mean *F*_ST_ value; and (b) a bootstrapping method developed by Whitlock and Guillaume (2009). This latter approach aims at comparing the observed difference between the overall *Q*_ST_ and the *F*_ST_ with the expected simulated distribution of this difference under a scenario of neutral evolution. We generated 100,000 bootstrap replicates of the expected *Q*_ST_–*F*_ST_ difference under the neutrality hypothesis for each trait, and built the corresponding distribution. In this approach, *p*‐values were estimated by assessing whether the observed value of the *Q*_ST_–*F*_ST_ difference overlapped its expected distribution under neutrality. We used the modification by Lind, Ingvarsson, Johansson, Hall, and Johansson (2011) of the approach of Whitlock and Guillaume (2009) to estimate the variance components of the simulated values of the *Q*_ST_–*F*_ST_ difference.

#### 2.7.4 Mantel tests

Mantel tests (Mantel, 1967) were used to analyse correlations between geographic distances, environmental distances (altitudinal), neutral genetic differentiation (population pairwise *F*_ST_), and quantitative genetic differentiation (population pairwise *Q*_ST_). They were run separately for each subspecies. First, a correlation test between population pairwise *F*_ST_ and population pairwise geographic distance matrices was performed to test for an isolation by distance relationship. Second, a correlation test between population pairwise *F*_ST_ and population pairwise *Q*_ST_ was performed for each trait to test if neutral genetic differentiation explained divergence in quantitative traits. Third, a correlation test between population pairwise *Q*_ST_ and population pairwise altitudinal differences was performed for each trait to test whether divergence in quantitative traits was related to altitudinal differences. Finally, we conducted partial Mantel tests to test for the association between population pairwise *Q*_ST_ and population pairwise altitude differences, while controlling for neutral genetic differentiation (*F*_ST_). All Mantel and partial Mantel tests were performed in R, with a significance threshold α = 0.05, using the vegan package (Oksanen et al., 2009).

## 3 RESULTS

### 3.1 Phenotypic differentiation between subspecies and populations

The two subspecies ‐ *A. m. pseudomajus* and *A. m. striatum* ‐ showed significant differences for several phenotypic traits (Table 1a; Figure S2). When grown in a common garden, plants of *A. m. pseudomajus* were on average taller, with more branches and nodes than plants of *A. m striatum*. However, both subspecies germinated on average at the same time, and showed similar internode length and SLA. Phenotypic differentiation between subspecies (ca. 1.9%) was lower than among populations (ca. 13.7%, see mean *R* ^2^ in Table 1a). For each subspecies, most traits showed phenotypic divergence among populations (see LRT in Table 1b). Germination date was the only trait that showed no significant difference among populations of *A. m. pseudomajus* (see LRT in Table 1b).

**TABLE 1:** Effects of subspecies and populations on phenotypic traits. (a) *R* ^2^ and *p*‐value from hierarchical generalized linear models (GLM) with subspecies alone and populations nested in subspecies. (b) Likelihood ratio tests (LRT) comparing the maximum‐likelihood fit between a model where populations were pooled and a model estimating the effect of the population of origin. A significant *p*‐value means the model including populations effect fitted the data better than the null model. Significant results (*p* < .05) are in bold

(a)	Subspecies		Populations in subspecies	
	*R* ^2^	*p*‐value	*R* ^2^	*p*‐value
Germination date	.0005	.968	.02	.260
Diameter	.003	.1719	**.05**	**.00068**
Nodes	**.045**	**<.0001**	**.19**	**<.0001**
Branches	**.032**	**<.0001**	**.10**	**.00001**
Plant height	**.041**	**<.0001**	**.26**	**<.0001**
Internode length	.003	.285	**.20**	**0**
SLA	.010	.052	**.137**	**.0003**
Mean	.019		**.137**	

(b)	*A. m. pseudmomajus*		*A. m. striatum*	
	LRT	*p*‐value	LRT	*p*‐value
Germination date	6	.570	12	**.021**
Diameter	17	**.001**	23	**<.0001**
Nodes	73	**<.0001**	27	**<.0001**
Branches	30	**<.0001**	19	**<.0001**
Plant height	32	**<.0001**	81	**<.0001**
Internode length	61	**<.0001**	24	**<.0001**
SLA	29	**.004**	28	**.004**

### 3.2 Neutral genetic differentiation

**FIGURE 2:**
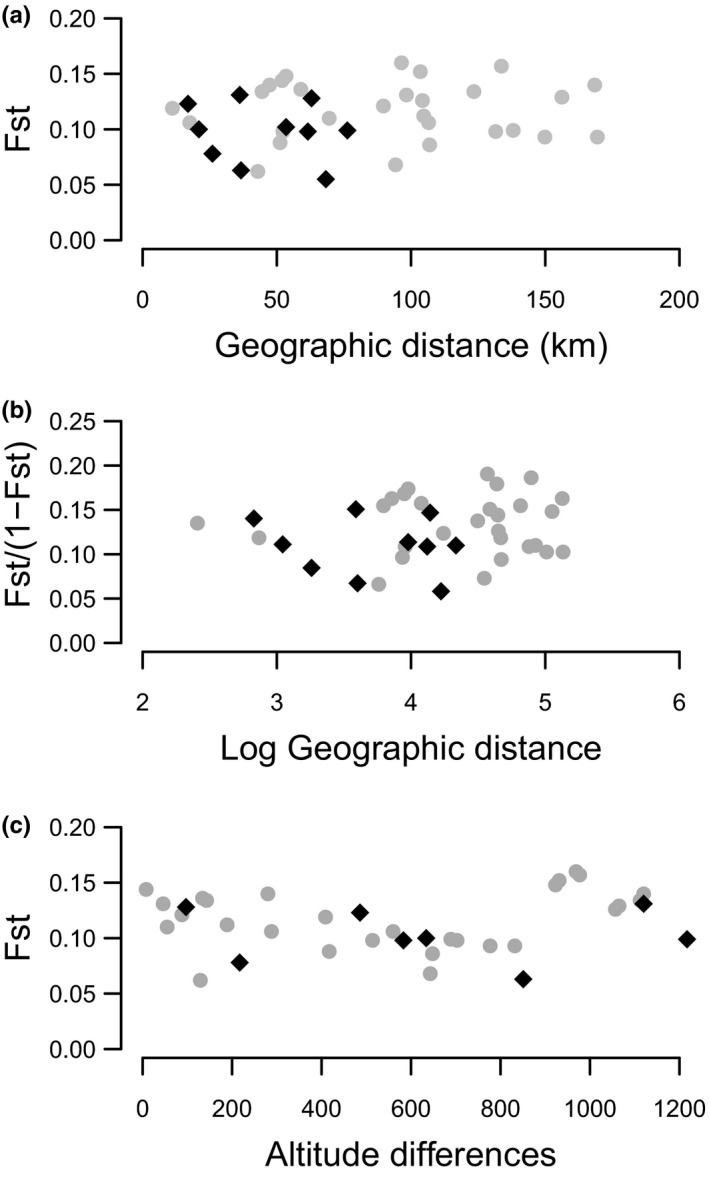
Pairwise neutral genetic differentiation *F*_ST_ plotted against pairwise geographic distances or altitudinal differences. Pairwise *F*_ST_ was estimated among eight *Antirrhinum majus pseudomajus* populations pairs (grey dots), and five *A. m. striatum* populations pairs (black diamonds). There were nonsignificant relationship between (a) *F*_ST_ and geographic distance in *A. m. pseudomajus* (Mantel *r* = .018, *p* = .46 ns) and in *A. m striatum* (Mantel *r* = −.15, *p* = .63 ns); (b) *F*_ST_/(1−*F*_ST_) and the log of geographic distance in *A. m. pseudomajus* (Mantel *r* = .04*, p* = .41 ns) and in *A. m striatum* (Mantel *r* = −.18, *p* = .6 ns); and (c) *F*_ST_ and altitude differences in *A. m. pseudomajus* (Mantel *r* = .23*, p* = .05 ns) and in *A. m striatum* (Mantel *r* = −0.3, *p* = .94 ns) [Colour figure can be viewed at wileyonlinelibrary.com]

Population neutral genetic differentiation was low but significant. Overall *F*_ST_ among populations of *A. m. pseudomajus* was 0.109 (*p* < .001), and ranged from 0.06 to 0.159 across population pairs (see Table S2, and see Pujol et al., 2017 for more details on population pairwise neutral genetic differentiation). *F*_ST_ among populations of *A. m. striatum* was 0.097 (*p* < .001), and ranged from 0.055 to 0.131 (Table S2). There was no significant relationship between population pairwise *F*_ST_ and population pairwise geographic distance, or between population pairwise *F*_ST_/(1−*F*_ST_) and the log of population pairwise geographic distance for either subspecies (Figure 2a,b, *F*_ST_ vs. distance: *A. m. pseudomajus* Mantel *r* = .018, *p* = .457, *A. m. striatum* Mantel *r* = −.15, *p* = .625, *F*_ST_/(1−*F*_ST_) vs. log distance: *A. m. pseudomajus* Mantel *r* = .04, *p* = .405, *A. m. striatum* Mantel *r* = −.18, *p* = .595). Similarly, there was no significant relationship between population pairwise *F*_ST_ and population pairwise altitude difference for either subspecies (Figure 2c), although Mantel tests showed a relationship close to significance levels in *A. m. pseudomajus* (*A. m. pseudomajus* Mantel *r* = .23, *p* = .052, *A. m. striatum* Mantel *r* = −.3, *p* = .943).

### 3.3 Changes in phenotypic traits with altitude

**FIGURE 3:**
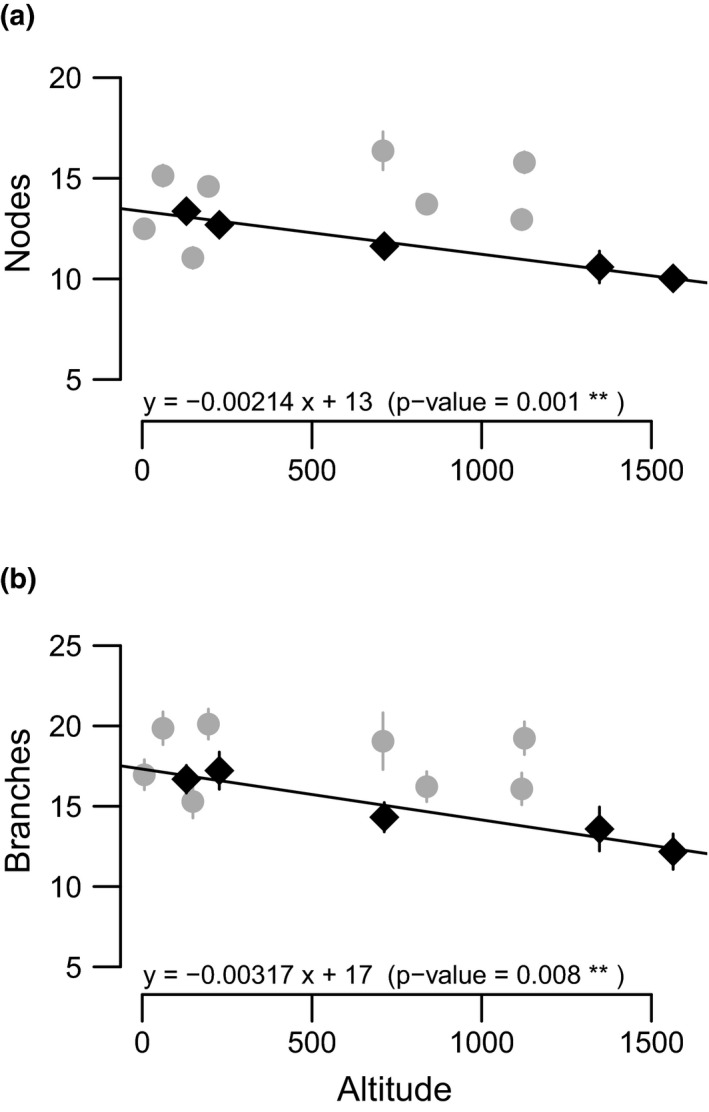
Trait changes with altitude. Population estimates of marginal means with standard errors of two phenotypic traits ((a) number of nodes, (b) number of branches) in populations of two subspecies of *Antirrhinum majus* grown in a common garden. Means are plotted against altitude of origin. Lines refer to the linear regression between trait mean estimates and altitude. Grey dots represent *A. m. pseudomajus* populations, black diamonds represent *A. m. striatum* populations. Equation of nonsignificant linear regressions were (a) y = 0.00125 x + 12 (*p* = .43 ns); and (b) y = −0.00048 x + 18 *(p* = .78 ns) for *A. m. pseudomajus* [Colour figure can be viewed at wileyonlinelibrary.com]

We found significant correlations between trait values (i.e., population estimates of marginal means) and altitude for two traits across *A. m. striatum* populations. Plants from populations at low altitude had more nodes and branches than plants from populations at high altitude for *A. m. striatum* (Figure 3, see population arithmetic means in Figure S2 and population estimates of marginal means for other traits in Figure S3). No phenotypic changes associated with altitude were significant in *A. m. pseudomajus*.

### 3.4 Inheritance of quantitative traits

Heritability estimates ranged between 0.11 and 0.83 for *A. m. pseudomajus*, and 0.01 and 0.89 for *A. m. striatum* (Table S3). Highest heritability estimates were for internode length in *A. m. pseudomajus* (0.83) and SLA in *A. m. striatum* (0.89). Several traits had similar heritabilities in each subspecies (stem diameter, number of nodes, internode length), as illustrated by their overlapping confidence intervals (CIs). However, this was not the case for other traits, i.e., there was no CI overlap (germination date, number of branches, plant height, SLA, Table S3).

### 3.5 *Q*_ST_–*F*_ST_ comparisons

**FIGURE 4:**
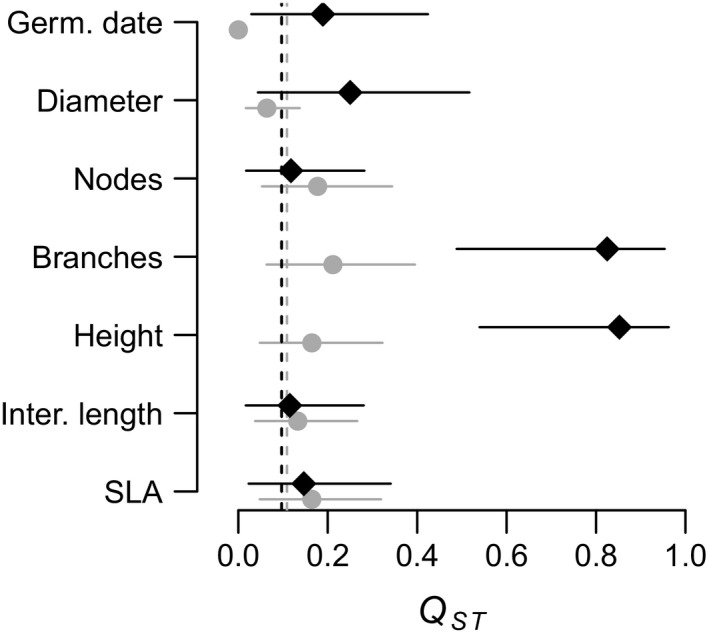
Overall *Q*_ST_ estimates with their 95% CI. Overall *Q*_ST_ estimates with their 95% CI are represented for seven phenotypic traits in eight *Antirrhinum majus pseudomajus* populations (grey dots) and five *A. m. striatum* populations (black diamonds) that were grown in a common garden. Average population *F*_ST_ is represented by the dashed grey line for *A. m. pseudomajus*, and the dashed black line for *A. m. striatum*. Branches, number of branches; Diameter, stem diameter; Germ.date, germination date; Height, plant height; Inter. Length, internodes length; Nodes, number of nodes; SLA, specific leaf area [Colour figure can be viewed at wileyonlinelibrary.com]

Overall *Q*_ST_ was not different from mean *F*_ST_ for *A. m. pseudomajus* traits (Figure 4a). In contrast, overall *Q*_ST_ was higher than mean *F*_ST_ for three traits in *A. m. striatum* as illustrated by their nonoverlapping CIs (number of branches, plant height and internode length, Figure 4b). Overall *Q*_ST_ was lower than mean *F*_ST_ for germination date in *A. m. pseudomajus* (Figure 4a). These results are fully consistent with results obtained via the bootstrapping method developed by Whitlock and Guillaume (2009). For one trait in *A. m. pseudomajus* (germination date), and for three traits in *A.m. striatum* (number of branches, plant height and internode length), observed values of overall *Q*_ST_–*F*_ST_ differences were either in the tail of the expected probability distribution under the hypothesis of neutrality, or did not overlap with this distribution (Figure S4 and S5).

In the present study, the average difference between overall *Q*_ST_–*F*_ST_ estimates was around 0.15, which is consistent with values found in the literature (around 0.12, see meta‐analysis from Leinonen, O'Hara, Cano, & Merilä, 2008). Yet, this difference reached 0.7 for the traits that we considered significant (traits with nonoverlapping *Q*_ST_ and *F*_ST_ CIs). This suggests that only traits with very high *Q*_ST_ values could be tested significant for the *Q*_ST_–*F*_ST_ difference in our study because the confident intervals were very large for most overall *Q*_ST_ estimates (Figure 4). This might be caused by lack of statistical power. This lack of statistical power might induce conservative results, with possible false negative overall *Q*_ST_–*F*_ST_ differences.

Mantel tests showed no relationship between population pairwise *Q*_ST_ and *F*_ST_ for most traits (Table 2). Only population pairwise *Q*_ST_ for germination date in *A. m. striatum* was significantly correlated with population pairwise *F*_ST_.

**TABLE 2:** Mantel tests and partial Mantel tests on pairwise *Q*_ST_ versus *F*_ST_ and *Q*_ST_ versus difference in altitude of origin (Alt. diff.), as well as partial Mantel tests on *Q*_ST_ versus Alt. diff. controlled for *F*_ST_, for phenology traits in (a) eight populations of *A. m. pseudomajus*; and (b) five populations of *A. m. striatum*, that were grown in a common garden. Significant values are indicated in bold

Traits	*Q*_ST_ versus *F*_ST_			*Q*_ST_ versus Alt. diff.				*Q*_ST_ versus Alt. diff./*F*_ST_		
	Mantel *r*		*p*‐value	Mantel *r*			*p*‐value	Mantel *r*		*p*‐value
(a) *A majus pseudomajus*										
Germination date	−.37	.937			−.13	.745			−.06	.602
Diameter	−.07	.618			−.19	.933			−.18	.780
Nodes	.07	.464			−.14	.795			−.16	.888
Branches	.11	.279			−.13	.713			−.16	.820
Height	.24	.187			−.15	.831			−.21	.911
Internode length	.20	.250			.05	.311			−.01	.442
SLA	.20	.246			.02	.379			−.04	.529
(b) *A majus striatum*										
Germination date	**.54**	**.042**			.05	.333			.27	.233
Diameter	.08	.366			−.09	.583			−.07	.566
Nodes	−.3	.825			**.93**	**.016**			**.92**	**.025**
Branches	−.23	.758			**.91**	**.025**			.91	.058
Height	−.61	.891			.09	.317			−.13	.6
Internode length	.36	.241			−.14	.667			−.04	.258
SLA	−.74	.950			.12	.300			−.17	.858

### 3.6 Increased quantitative genetic differentiation with altitude difference

**FIGURE 5:**
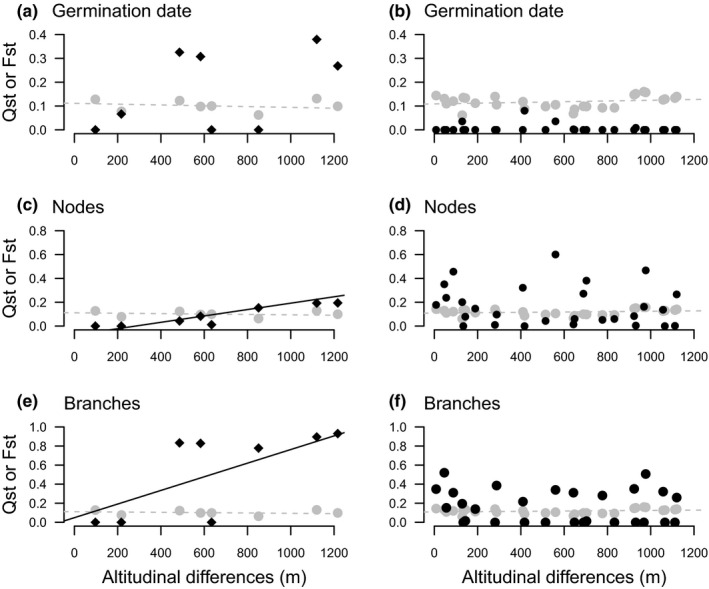
Illustration of the relationship between population pairwise *Q*_ST_, *F*_ST_ and altitudinal differences. Population pairwise quantitative trait differentiation (*Q*_ST_) for the germination date, the number of branches and the number of nodes in *Antirrhinum majus striatum* (a, c and e, black diamonds) and *A. m. pseudomajus* (b, d and f, black dots). Black lines are only indicative. They represent the informal linear relationship of population pairwise *Q*_ST_ on population pairwise altitudinal differences (m), thereby illustrating the statistical dependency formally tested by Mantel test approaches. Grey dots and dashed line refer to population neutral genetic differentiation (*F*_ST_) [Colour figure can be viewed at wileyonlinelibrary.com]

Mantel tests showed a significant correlation between population pairwise *Q*_ST_ and population pairwise altitudinal differences for two traits in *A. m. striatum*: the number of nodes and the number of branches (Table 2, Figure 5). For both traits, the increase in pairwise population differentiation associated with an increase in altitudinal difference was higher for the *Q*_ST_ than for the *F*_ST_ (Figure 5c,e). Partial mantel tests showed that population pairwise *Q*_ST_ was significantly correlated with differences in altitude for number of nodes (and marginally significant for number of branches, see *Q*_ST_ vs. Alt. diff./*F*_ST_ in Table 2) while controlling for neutral genetic differentiation (*F*_ST_). This result is expected under the hypothesis that the divergence among populations of *A. m. striatum* in the number of nodes is a result of altitude‐mediated divergent selection. In contrast, none of the seven traits showed a significant correlation between population pairwise *Q*_ST_ and population pairwise altitude difference in *A. m. pseudomajus*.

## 4 DISCUSSION

Our results support the hypothesis of differential adaptation between *A. m. pseudomajus* and *A. m. striatum* subspecies. We detected phenotypic differentiation in a common garden among populations of *A. m pseudomajus*, among populations of *A. m striatum*, and among subspecies. For both subspecies, local adaptation and neutral evolution explained the extent to which populations diverged over their geographic range, with slight differences between subspecies. Signatures of potential selection were found for few traits. Potential divergence along altitude was also detected, but only for one subspecies: *A. m. striatum*.

Our findings support the idea that *Q*_ST_–*F*_ST_ comparisons are a good first step for exploring the potential roles of divergent natural selection and neutral evolutionary processes in phenotypic divergence (Edelaar et al., 2011; Ovaskainen et al., 2011; Whitlock, 2008; Whitlock & Gilbert, 2012). They highlighted how traits can be used to identify the potential ecological pressures underlying natural selection, with some traits potentially involved with *A. majus* adaptation to the conditions of populations' local sites of origin, and a subsample of these traits potentially playing a role in *A. m. striatum* adaptation to altitude.

### 4.1 Adaptive evolution of *A. m. striatum* populations along the altitudinal gradient

Our results imply that the quantitative genetic basis of two of the seven traits studied (number of nodes, and the marginally significant number of branches) was shaped by divergent selection between populations from different altitudes in *A. m. striatum* but not in *A. m. pseudomajus*. Most studies on plant adaptation to altitude report the selection of smaller plants at higher altitudes (Halbritter et al., 2018; Körner, 1999). In agreement with this expectation, we found that *A. m. striatum* plants at higher altitudes had fewer branches and fewer nodes. It is important to note that branches can only grow from axillary buds located between leaf and stem at the level of nodes. These two developmentally correlated traits can reflect the same growth measurement. Their lack of independence is therefore not surprising. Although evidence for changes in leaf traits with elevation can be found in the literature (Halbritter et al., 2018; Read et al., 2014), our results did not support a potential scenario of selection based on SLA in *A. m. striatum*.

### 4.2 Support for different subspecies scenarios of adaptation to local sites of origin

Our results showed that quantitative genetic differentiation was higher than what could be explained by neutral evolutionary divergence among *A. m. striatum* populations for three of the seven traits (number of branches, plant height and internode length). They imply that adaptation to local sites of origin potentially shaped the phenotypic diversity of populations for *A. m. striatum* across their geographic range. We used classical overall *Q*_ST_–*F*_ST_ comparisons to detect potential adaptation to local sites conditions (reviewed in Leinonen et al., 2013) and also more recent methods to insure that our findings were robust against a range of neutral evolution scenarios for these traits (Whitlock, 2008). Furthermore, our approach minimized the possibility that phenotypic differences between populations were generated by environmental effects by using a common garden experiment, and including trait heritability estimates in *Q*_ST_ calculations (Pujol, Salager, et al., 2008; Pujol, Wilson, et al., 2008; Spitze, 1993). In contrast, four of the seven studied traits (germination date, diameter, number of nodes and SLA) did not show departure from plausible baseline scenarios of neutral evolutionary divergence when using overall *Q*_ST_–*F*_ST_ comparisons. One particular trait (germination date) was in fact more similar among populations than expected under neutrality in *A. m. pseudomajus*. A scenario of stabilizing selection is classically extrapolated in the case of similar results (Lamy, Plomion, Kremer, & Delzon, 2012) but another plausible explanation is that population similarity might have been caused by convergent phenotypic responses to the common garden environmental similarity. Here we found different patterns between subspecies, which supports the hypothesis of their potential adaptive divergence. Caution must nevertheless be exercised when interpreting different *Q*_ST_–*F*_ST_ patterns between subspecies as the signature of different adaptive processes. Our results cannot be interpreted as direct proof, but only as evidence that potentially favours this hypothesis.

### 4.3 The ecological significance of adaptation to local sites of origin in *A. majus*

In the absence of environmental measures included in the overall *Q*_ST_–*F*_ST_ analysis, it is impossible to identify the environmental agents of local selection that shape the quantitative genetic variation of traits. The functions behind the divergent traits can nevertheless be used to discuss plausible evolutionary scenarios of natural selection. Our results imply that adaptation to local sites of origin has potentially shaped the vegetative architecture of plants that is specific to each *A. majus* population. The quantitative genetic variation of several phenotypic traits characterising the vegetative growth and development of plants (plant height, internode length, number of branches) has probably diverged among populations as a result of adaptation to local sites of origin. Divergence in the genetic variation underlying the shape and size of plants was previously found at the level of *Antirrhinum* species, though its adaptive significance was not tested (Langlade et al., 2005). In southern France and northern Spain experiencing a Mediterranean climate, dryer locations are expected to select for plants with a bushier vegetative architecture, i.e., plants with smaller leaves and more branches that have better water use efficiency and resilience to drought stress (Langlade et al., 2005). It is difficult to identify exactly which environmental pressures underlie selection at local sites because several combinations of environmental parameters (vegetation cover, wind, disturbance, temperature, water availability, etc) can interact to affect phenotypic traits.

### 4.4 Gene flow, ecological and reproductive isolation

Our findings imply that the most likely evolutionary scenario applying to *A. majus* requires invoking a history of adaptation to local sites in a complex background of gene flow, ecological heterogeneity and reproductive isolation. The Pyrenees are widely acknowledged to constitute a heterogeneous landscape that promotes complex patterns of population connectivity and is prone to generate local adaptation (Alberto et al., 2010). Our *Q*_ST_–*F*_ST_ comparisons reflected a potential scenario of population divergent adaptation to contrasting environmental conditions between local sites of origins. Our findings also suggested that evolutionary signatures of local adaptation differed between *A. m. pseudomajus* and *A. m striatum*, as illustrated by the potential adaptation to altitude detected only for *A. m. striatum*. One might speculate that this divergence might be related to the distribution of *A. m. striatum* populations across a narrower range of climatic conditions, even if both subspecies share to a large extent the same ecological niche (Khimoun et al., 2013). However, caution must be exercised with this explanation because the state of the environment in the past, when divergence might have occurred, is unknown and might have differed from that at present. Contrasting hypotheses might be interesting to consider, e.g., different evolutionary potentials in the presence of similar environmental pressures. These scenarios are not exclusive and can reinforce each other through a feedback loop between reproductive isolation, neutral divergence and selection.

Restricted gene flow or strong selection pressures are required for evolutionary divergence. Genetic drift, or foundation events by different gene pools, might have shaped differentially the genetic background of *A. majus* populations and to some extent subspecies at the scale of their global geographic range. There is evidence for the genetic signature of restricted gene exchanges in *A. majus* (Pujol et al., 2017). No genetic isolation by distance was found, but ecological barriers characterizing the mountain landscape of the Pyrenees probably participate to isolate populations (Pujol et al., 2017). At first sight, *A. majus* subspecies' divergence might not be expected because both subspecies are interfertile (Andalo et al., 2010), and no genome wide barrier to gene flow was found between them at the scale of a hybrid zone across c. 2 km in the Pyrenees (Ringbauer et al., 2018). There is also evidence for gene exchanges between the two subspecies in several contact zone locations at the periphery of their geographic ranges (Khimoun et al., 2011). Yet, subspecies' flower colour differences attest that flower colour genes are under frequency dependent selection and generate reproductive isolation between subspecies (Ringbauer et al., 2018; Tastard et al., 2012). This reproductive isolation might contribute to the subspecies phenotypic divergence of the other traits that we detected here.

In conclusion, our findings corroborate the utility of *Q*_ST_–*F*_ST_ approaches conducted in common garden experiments to explore potential adaptive evolutionary divergence among populations and between subspecies in plants. They also illustrate the limits of this approach that identifies traits that might be involved with local adaptation but which fails to provide direct evidence for their response to selection. Here, our common garden results for *A. m. pseudomajus* and *A. m. striatum* populations identified vegetative traits that might play a role in the local adaptation and the differential adaptation of *A. m. pseudomajus* and *A. m. striatum* along altitudinal gradients. They suggest that the adaptation to climate variables of these otherwise interfertile subspecies might differ as a result of reproductive isolation.

## AUTHOR CONTRIBUTIONS

BP designed the research program. SM, JA, VB, ML carried out the experiments; AG and SM analysed the data; AG, SM and BP wrote the manuscript.

## Supporting information

Supplementary Material

## Data Availability

All the data sets (phenotypic data, molecular data and metadata input files) used in the analyses presented in this article and the R code protocols used to run these analyses with these data sets are already freely accessible on the ZENODO [Open Aire EU official] repository: https://doi.org/10.5281/zenodo.3628168
