# Supplementary material for: Potential adaptive divergence between subspecies and populations of snapdragon plants inferred from Q ST–F ST comparisons
Source: Mol Ecol. 2020 Jul 24;29(16):3010–21. doi: 10.1111/mec.15546 (PMC7540467; doi:10.1111/mec.15546)
Supplement: Supplementary file 1 — Supplementary Material [file MEC-29-3010-s001.pdf]

MOLECULAR ECOLOGY

Supplemental Information for:

Potential adaptive divergence between subspecies and populations of snapdragon plants inferred from Q_{ST} – F_{ST} comparisons

Sara Marin, Anaïs Gibert, Juliette Archambeau, Vincent Bonhomme, Mylène Lascoste and Benoit Pujol

Table of Contents:

**Supplementary information**	Page 2
**Table S1**	Page 3
**Table S2**	Page 4
**Table S3**	Page 5
**Figure S1**	Page 6
**Figure S2**	Page 7
**Figure S3**	Page 8
**Figure S4**	Page 9
**Figure S5**	Page 10

MOLECULAR ECOLOGY

Supplementary information

The variance in altitude was not significantly different between subspecies, and should not drive difference between taxa. Indeed, we performed an ANOVA testing the difference of variance in altitude between subspecies and we found no significant differences (P-value = 0.4, F-value = 0.765, df =1, Sum Sq =225889, Mean Sq= 225889, df Residuals = 11, Sum Sq residuals= 3247238, Mean Sq Residuals= 295203).

MOLECULAR ECOLOGY

TABLE S1 Description of *Anthirinum majus* populations grown in the common garden experiment. *Nfam*= number of families, *N*= number of plants

Acronym	Lat	Long	Location	Elevation (m)	Subspecies	Description	*Nfam*	*N*
BAG	43.10	2.98	Bages	6	*pseudomajus*	Dunes on seaside (rocky / herbaceous)	40	67
BAN	42.49	3.12	Banyuls-sur-Mer	61	*pseudomajus*	Rockside bank (rocky)	32	54
THU	42.64	2.72	Thuir	130	*striatum*	Roadside bank (herbaceous)	34	60
LAG	43.09	2.58	Lagrasse	149	*pseudomajus*	Roadside bank (rocky / herbaceous)	32	55
BES	42.21	2.67	Besalú	195	*pseudomajus*	Stone walls in village	39	69
LUC	42.97	2.26	Luc-sur-Aude	227	*striatum*	Roadside bank and river-side bank (rocky)	19	29
RIP	42.21	2.20	Ripoll	709	*pseudomajus*	Roadside bank (herbaceous)	9	16
LYS	42.83	2.20	'Pierre-Lys' gorge	713	*striatum*	Roadside bank (rocky / herbaceous)	32	53
CAL	42.10	1.83	Berga	838	*pseudomajus*	Roadside bank (herbaceous)	42	69
PAR	42.31	2.20	Pardines	1118	*pseudomajus*	Roadside bank (herbaceous)	32	58
SAL	42.23	1.74	Saldes	1126	*pseudomajus*	Banks in pasture (herbaceous)	30	55
MIJ	42.73	2.04	Mijanès	1347	*striatum*	Roadside bank (herbaceous)	10	18
MON	42.51	2.12	Mont-Louis citadelle	1564	*striatum*	Stone walls on fortifications	21	34
						All populations	*372*	*637*

MOLECULAR ECOLOGY

TABLE S2 Population pairwise F_{ST} for a) *Anthirinum majus pseudomajus* and b) *A. m. striatum.*

a) *Anthirinum majus pseudomajus*

Obs.	BAG	BAN	BES	CAL	LAG	PAR	RIP	SAL
BAG	0.000000	0.109661	0.111560	0.093282	0.134302	0.134404	0.097873	0.140489
BAN	0.109661	0.000000	0.136375	0.093343	0.120889	0.125510	0.086353	0.128994
BES	0.111560	0.136375	0.000000	0.068186	0.131472	0.147507	0.098488	0.152424
CAL	0.093282	0.093343	0.068186	0.000000	0.099317	0.139897	0.062174	0.106419
LAG	0.134302	0.120889	0.131472	0.099317	0.000000	0.159517	0.106148	0.156761
PAR	0.134404	0.125510	0.147507	0.139897	0.159517	0.000000	0.119122	0.144271
RIP	0.097873	0.086353	0.098488	0.062174	0.106148	0.119122	0.000000	0.088219
SAL	0.140489	0.128994	0.152424	0.106419	0.156761	0.144271	0.088219	0.000000

b) *Anthirinum majus striatum*

Obs	LUC	LYS	MIJ	MON	THU
LUC	0.000	0.123	0.131	0.102	0.128
LYS	0.123	0.000	0.100	0.063	0.098
MIJ	0.131	0.100	0.000	0.078	0.099
MON	0.102	0.063	0.078	0.000	0.055
THU	0.128	0.098	0.099	0.055	0.000

MOLECULAR ECOLOGY

TABLE S3 Quantitative genetics parameters for phenotypic traits among eight populations of *Anthirinum majus pseudomajus* and five populations of *Anthirinum majus striatum* grown in a common garden. Values for trait heritability (h^2), family variance (Vw), among-population variance (Vb), residual variance corresponding to the within-population variance (Vres) and quantitative trait divergence (Q_{ST}). The degrees of freedom used in the bootstrapping procedures are seven for the among-population component (Vb) for *A.m. pseudomajus* and four for *A. m. striatum*. Degrees of freedom are given in this table for the within-population component (df$V$w).

Traits	h^2	h^2.CI	Vw	Vb	Vres	df$V$w	Q_{ST}	Q_{ST}
a) *A. m. pseudomajus*								
Germination date	0.35	0.3; 0.43	14.05	0	65.13	184	0	0
Diameter	0.19	0.16; 0.23	0.07	0.02	0.68	184	0.06	0.06
Nodes	0.45	0.38; 0.53	2.97.	2.69	10.27	184	0.19	0.19
Branches	0.11	0.09; 0.14	2.46	2.80	41.25	184	0.22	0.22
Plant height	0.32	0.27; 0.38	19.72	16.1	105.45	184	0.17	0.17
Internode length	0.83	0.73; 0.94	0.10	0.06	0.14	184	0.14	0.14
SLA	0.25	0.21; 0.30	172	141.7	1216	184	0.17	0.17
b) *A. m. striatum*								
Germination date	0.12	0.09; 0.16	4.37	4.49	71.98	82	0.20	0.20
Diameter	0.17	0.12; 0.23	0.05	0.07	0.55	82	0.27	0.27
Nodes	0.56	0.44; 0.70	2.90	1.64	7.57	82	0.12	0.12
Branches	0.01	0.01; 0.01	0.12	3.52	26.31	82	0.88	0.88
Plant height	0.06	0.04; 0.08	2.47	92.95	84	82	0.90	0.90
Internode length	0.81	0.66; 0.98	0.08	0.04	0.11	82	0.12	0.12
SLA	0.89	0.73; 1	541	400	689	82	0.16	0.16

MOLECULAR ECOLOGY

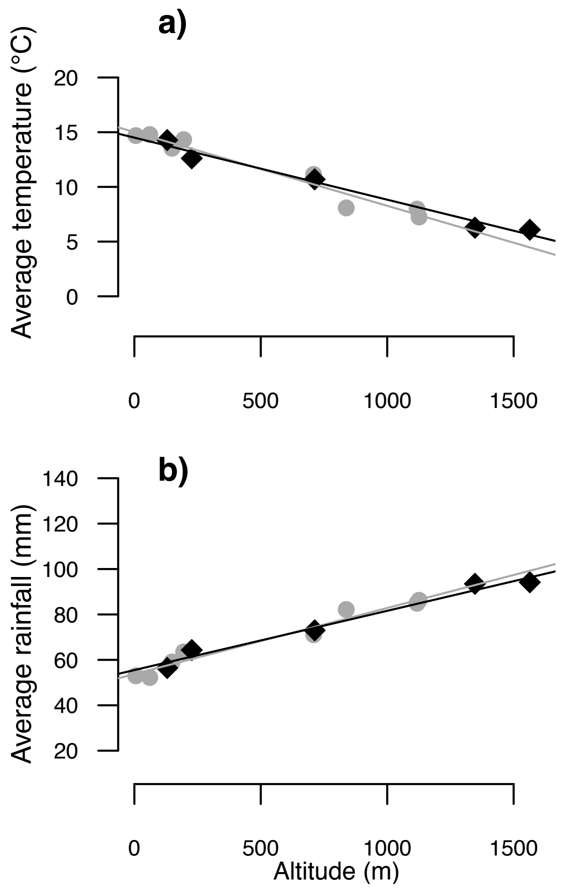

FIGURE S1.
Annual average temperatures and rainfall of eight *Antirrhinum majus pseudomajus* populations (grey dots) and five *A. m. striatum* populations (black diamonds) from the Southern France. Population average temperature (a) and average rainfall (b) as a function of altitude. Bioclimatic data was extracted from the *WorldClim* database (www.worldclim.org).

MOLECULAR ECOLOGY

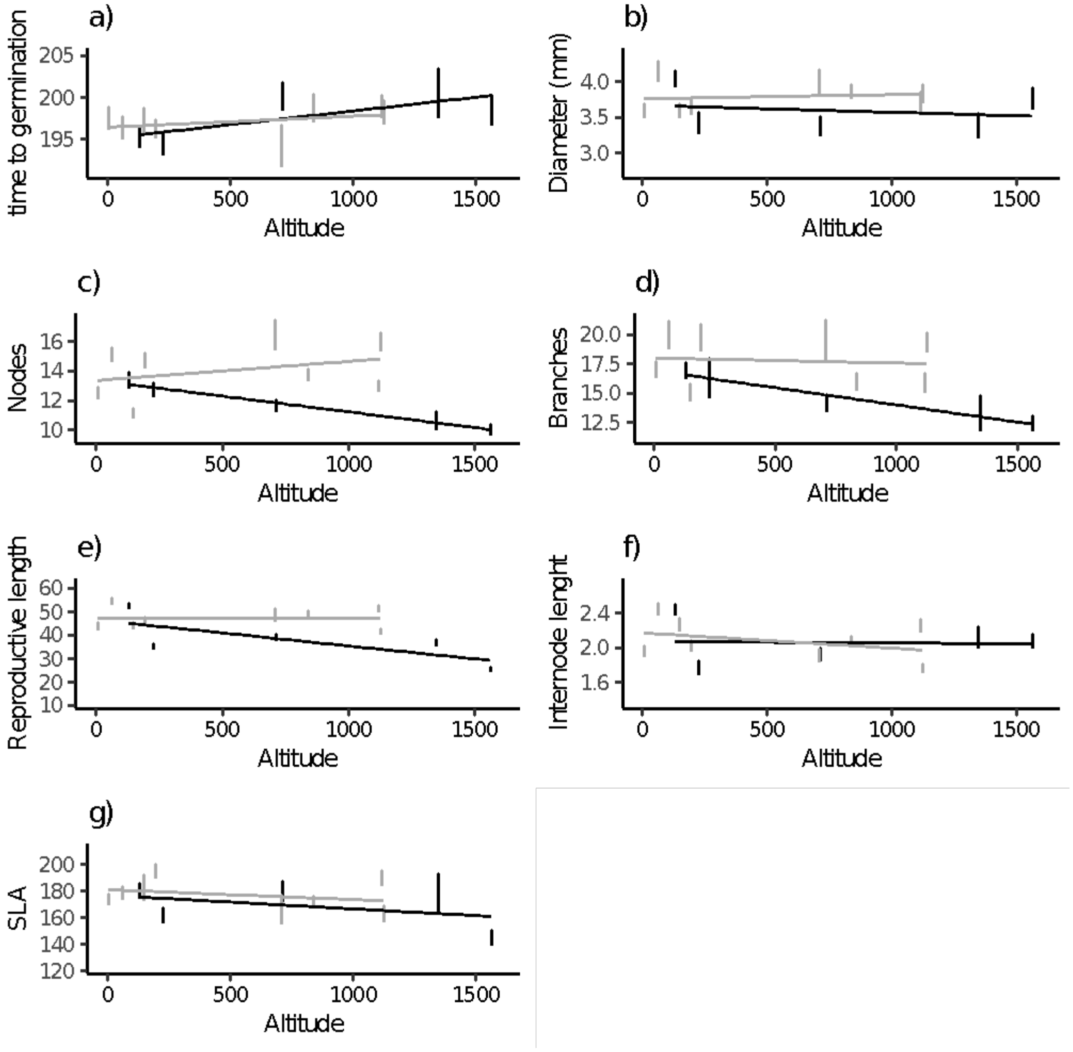

FIGURE S2.

Population arithmetic means with standard errors of seven phenotypic traits in populations of two subspecies of *Antirrhinum majus* grown in a common garden. Means are plotted against altitude of origin. Grey dots represent *A. m.* ssp. *pseudomajus* populations, black diamonds represent *A. m.* ssp. *striatum* populations.

MOLECULAR ECOLOGY

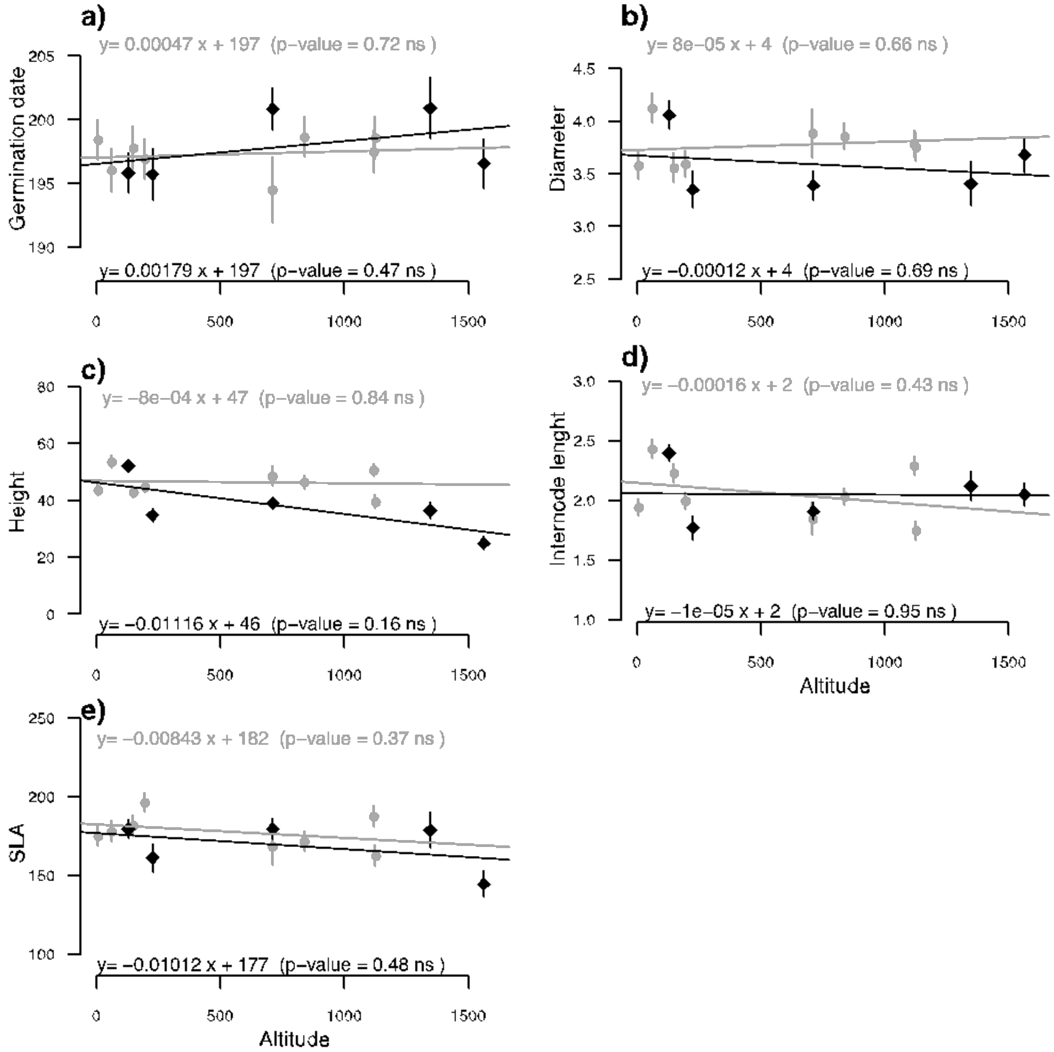

FIGURE S3. Population estimates of marginal means with standard errors of five phenotypic traits in populations of two subspecies of *Antirrhinum majus* grown in a common garden. Means are plotted against altitude of origin. Lines refer to the linear regression between traits means estimates and altitude. Grey dots and lines represent *A. m. pseudomajus* populations, black diamonds and lines represent *A. m.* ssp. *striatum* populations.

MOLECULAR ECOLOGY

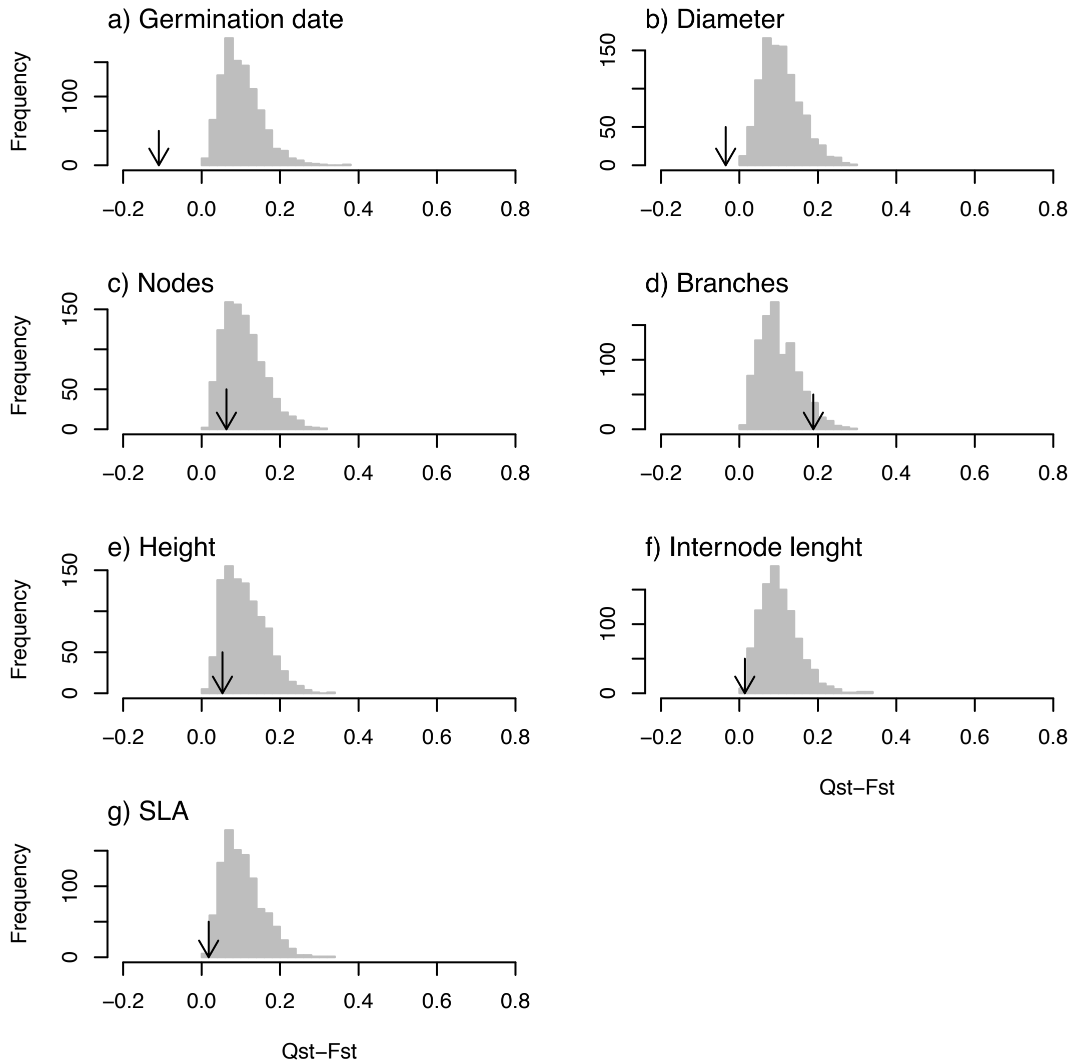

FIGURE S4. The simulated distribution of Q_{ST} - F_{ST} for a neutral trait, and the observed point estimates of Q_{ST} - F_{ST} differences in seven phenotypic traits measured for the eight *Antirrhinum majus pseudomajus* populations from the Southern France. The distribution of Q_{ST} - F_{ST} differences for a neutrally evolving trait was simulated following Whitlock and Guillaume (2009) based upon the observed population differentiation in neutral markers (F_{ST}) and the within-population variance in each trait. The arrow indicates the observed Q_{ST} - $F_{ST.}$

MOLECULAR ECOLOGY

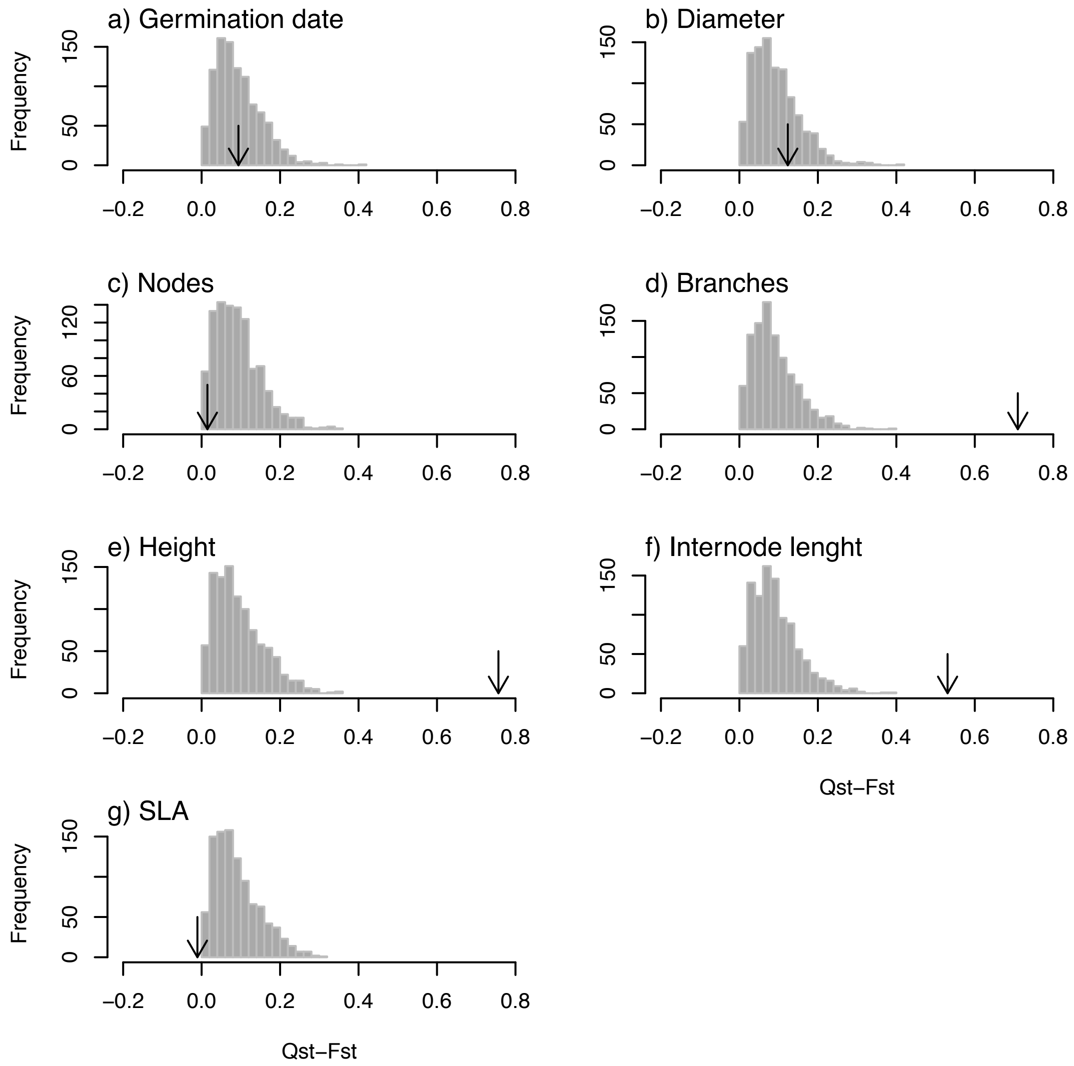

FIGURE S5. The simulated distribution of Q_{ST} - F_{ST} for a neutral trait, and the observed point estimates of Q_{ST} - F_{ST} differences in seven phenotypic traits measured for the five *Antirrhinum majus striatum* populations from the Southern France. The distribution of Q_{ST} - F_{ST} differences for a neutrally evolving trait was simulated following Whitlock and Guillaume (2009) based upon the observed population differentiation in neutral markers (F_{ST}) and the within-population variance in each trait. The arrow indicates the observed Q_{ST} - $F_{ST.}$
